# Investigation of the Impact of Infrared Sensors on Core Body Temperature Monitoring by Comparing Measurement Sites

**DOI:** 10.3390/s20102885

**Published:** 2020-05-19

**Authors:** Hsuan-Yu Chen, Andrew Chen, Chiachung Chen

**Affiliations:** 1 Department of Materials Science and Engineering, University of California, San Diego, CA 92093, USA; wakaharu37@gmail.com; 2 Africa Research Center, National Chung Hsing University, Taichung 40227, Taiwan; b95501060@ntu.edu.tw; 3 Department of Bio-industrial Mechatronics Engineering, National ChungHsing University, Taichung 40227, Taiwan

**Keywords:** body temperature, COVID-19, infrared thermometer, forehead temperature, tympanic temperature

## Abstract

Many types of thermometers have been developed to measure body temperature. Infrared thermometers (IRT) are fast, convenient and ease to use. Two types of infrared thermometers are uses to measure body temperature: tympanic and forehead. With the spread of COVID-19 coronavirus, forehead temperature measurement is used widely to screen people for the illness. The performance of this type of device and the criteria for screening are worth studying. This study evaluated the performance of two types of tympanic infrared thermometers and an industrial infrared thermometer. The results showed that these infrared thermometers provide good precision. A fixed offset between tympanic and forehead temperature were found. The measurement values for wrist temperature show significant offsets with the tympanic temperature and cannot be used to screen fevers. The standard operating procedure (SOP) for the measurement of body temperature using an infrared thermometer was proposed. The suggestion threshold for the forehead temperature is 36 °C for screening of fever. The body temperature of a person who is possibly ill is then measured using a tympanic infrared thermometer for the purpose of a double check.

## 1. Introduction

Body temperature is an indicator of human physiological activity and health, especially in pediatrics, surgery and general emergency departments [1].

Most of the early methods of measuring body temperature used contact mercury thermometers. Advances in electrical technology mean that contact-type electronic thermometers have become widely used. The measurement sites are the sublingual mouth, the rectum, the axillary and the base of the urethra. Some medical reports show that contact thermometers accurately measure body temperature [2,3]. To screen for an illness, the body temperature of many individuals must be measured, so noncontact type infrared thermometers (IRT) are used to measure the tympanic (ear) and forehead temperature. These IRTs are fast, convenient and safe to use. Medical IRTs for ear temperature and forehead temperature are used in medicine.

When the temperature of a natural object is higher than the absolute temperature, the surface emits thermal radiation. The infrared thermometer (IRT) detects this radiant energy that is released by the object via sensing elements and converts it into an electrical signal. After signal processing, the measured temperature is displayed on the IRT. This principle is used to develop an IRT to measure body temperature [4,5,6,7].

In 2003, severe acute respiratory syndrome, which is commonly known as SARS, was prevalent in Asia and caused panic in many countries. IRT is a rapid detection method and the most commonly used IRTs measure the temperature of the tympanic and the forehead. The tympanic is close to the core temperature of the body and gives the most accurate representation of the body temperature [4,6,7,8,9,10]. The screening of passengers for signs of Ebola infection was observed with a noncontact thermometer at five airports in USA in 2014 [11].

IRTs are used to measure the tympanic or forehead temperatures. The probe cover must be changed, and the probe must be adjusted to fit the shape of the ear canal, so measuring the tympanic temperature is a troublesome process. A forehead IRT is convenient and noninvasive, so any risk of mutual infection is prevented, and as such, public places, such as airports, schools, hospital, and transportation vehicles, used this type of device to screen the public in the SARS era [12,13,14]. This method is also currently used to prevent the spread of the coronavirus (COVID-19). However, noncontact IRTs are not proven in terms of accuracy, so it is necessary to determine whether an IRT gives as reliable information as other thermometers. The performance of forehead thermometers has been questioned [15]. Hsiao et al. [16] suggested taking temperature measurements twice for people before entering the hospital to reduce the risk of COVID-19 spread.

The surface emissivity of an object has the most significant effect on the temperature that is measured by an IRT. The emissivity of the tympanic membrane is assumed to be 1.0, but the emissivity of human skin is between 0.976 and 0.984 [17,18,19,20].

There are two types of infrared techniques available for the surface temperature detection. One is the point estimation to detect the tympanic temperature or the forehead temperature, and the device is called tympanic IRT or forehead IRT. The other is the IR thermography or thermal imaging. The thermal detector is used to detect the receive radiation from the targets. The sensing elements of thermal detectors are the pyroelectric detector and the thermopile for infrared thermometers and the bolometer focal plane arrays (Bolometer FDA) for IR thermography.

Recently, research on the application of infrared thermography on human body temperature measurement has been carried out. Sharma and Yadav [21] proposed a noncontact temperature algorithm for face detection in a video sequence and validated its performance against standard temperature device. Rodriguez-Lozand et al. [22] proposed a novel method to segment the forehead region of the human beings and to calculate the mean temperature of these area. With this simple and accurate method, the thermal face images in different features are presented. Chaglla et al. [23] introduced an ear-based device to measure the ear temperature continuously. The novel design involved coating graphene platelets on the lens of an infrared thermopile sensor, and the performance of this device was validated by comparing it with others commercial ear thermometers. Tay et al. [24] evaluated three types of infrared thermal detection systems for fever screening in tropical conditions and found that the devices with video monitoring had very high specificity and the handheld thermograph could not be used for fever screening.

Many factors affect the temperature measurement of the human body [25,26]. Erickson and Kirklin [27] performed invasive measurements using pulmonary artery temperature as an indicator to compare tympanic, bladder, oral and axillary temperature. The difference between the pulmonary artery temperature and the ear temperature was 0.07 °C ± 0.41 °C, for the bladder temperature, the value was 0.03 °C ± 0.23 °C, for the oral temperature, the value was 0.05 °C ± 0.26 °C and for the axillary temperature, the value was −0.68 °C ± 0.57 °C. These results show that the bladder temperature and the mouth temperature are closest to the pulmonary artery temperature, followed by the tympanic temperature, and the value for the axillary temperature was low.

Patel et al. [28] compared the correlation between esophageal temperature, tympanic temperature and forehead surface temperature. The difference between the temperature of the esophagus and the frontal temperature was −1.64 ° C to 2.32 °C, for the ear temperature, the difference was −1.02 °C to 0.74 °C and for the tympanic temperature and forehead temperature, the difference was −1.48 °C to 2.52 °C. The correlation coefficients for the forehead temperature, the esophageal temperature, and ear temperature are small.

Measuring the temperature of the forehand using an IRT is convenient, but some studies show that the measurements are not accurate. There are three types of forehead thermometer: a thermistor probe, a liquid crystal strip and an IR thermometer. The thermistor probe and the liquid crystal strip are contact-type thermometers. The thermistor probe is a deep body thermometer because it is inserted into the tissue of the forehead.

Studies show that a contact-type deep skin forehead thermometer is suited to clinical use [29,30,31,32,33]. This device was very accurate. Duran et al. [8] compared the performance of a contact-type forehead thermometer with that of an axillary glass–mercury thermometer and found that both thermometers give similar readings. Zeiner et al. [34] compared the body temperature that was measured using a deep forehead thermometer and a bladder thermometer and found no significant difference between the two readings. Liquid crystal thermometers have been used to measure forehead temperature [35,36,37,38,39]. These studies show that liquid crystal devices are not as accurate as forehead thermistor thermometers and do not give as accurate a reading for core temperature as a rectal or tympanic measurement.

Patel et al. [27] compared esophageal, tympanic and forehead temperature for adults. The mean difference between the esophageal and the forehead temperature was 0.3 °C and the mean difference between the tympanic and the forehead temperature was 0.5 °C. The forehead temperature was measured using a contact-type, liquid crystalline thermometry strip. The authors noted that the forehead temperature was not an accurate representation of the standard core temperature. Asadian et al. [40] measured the oral, axillary, tympanic and forehead temperature. The central nasopharyngeal measurement was used as a standard value. The results show that the forehead and tympanic readings were the least and most accurate, respectively. The forehead temperature was measured using a contact-type strip thermometer. Berksoy et al. [41] used a noncontact IRT to measure forehand temperature and compared the reading with those for tympanic and rectal temperature. The results show that measurements of the tympanic temperature are more practical than axillary thermometry.

Duncan et al. [42] showed that the difference between the oral temperature and the temperature that was recorded using an IRT was 0.89 °C ± 0.58 °C. The authors commented that the thermometers were not interchangeable. Kocoglu et al. [10] compared the accuracy of a tympanic IRT reading with the readings for axillary and rectal temperature that were measured using a glass thermometer. The highest temperature was the rectal temperature and the lowest temperature was the axillary temperature. The difference between the mean values for tympanic and rectal temperature was <0.2 °C.

Yeoh et al. [43] studied the tympanic membrane vicinity as the measurement site of core body temperature and found the mean temperature of rectum, esophagus, left ear and right ears were 37.2 °C, 36.8 °C, 36.2 °C and 36.1 °C, respectively. Oguz et al. [44] measured the tympanic and axillary body temperature of 1364 children and found the mean axillary and tympanic temperatures were 36.04 °C and 36.91 °C, respectively. The axillary and tympanic temperature that are considered as fever are >37.0 °C and >37.8 °C, respectively. Cutuli et al. [45] reported the inconsistent opinions of the unit protocol for measuring body temperature and suggested the requirement of detailed studies of the noninvasive temperature measurement methods.

Mogensen et al. [46] used the rectal temperature as a standard value. The study measured the tympanic temperature using an IRT and the forehead temperature using a contact-type temporal thermometer. The results show that the tympanic measurement was adequate for screening purposes but the forehead temperature was not. Kistemaker et al. [47] tested two types of contact forehead thermometers and compared the readings with those for a rectal thermistor. The results showed that a contact-type forehead thermometer registered a higher temperature than a rectal thermistor. Dante et al. [48] compared the performance for contact-type forehead, tympanic and axillary thermometers. The mean value for tympanic temperature was higher than that for the forehead and axillary thermometers, and these devices were not interchangeable. Ng et al. [13] used a noncontact, handheld IRT to measure the forehead skin temperature. The reference temperature was measured using an electronic thermometer. The surface temperature was underestimated by 2 °C, so a temperature of 35.6 °C was the index temperature for fever if the forehead temperature was measured using a noncontact, handheld IRT. Ng et al. [14] studied the relationship between the facial skin temperature that was detected by an IRT, and the actual temperature was detected using a direct thermometer. They recommend that the measured temperature of 35.5 °C signified a fever.

Measuring body temperature is an effective technique for screening SARS and other flus. The coronavirus (COVID-19) has spread globally and temperature measurement is used to rapidly screen people. Handheld forehead IRTs are easy to use, rapid, noncontact and inexpensive, so they are widely used. This IRTs was reported to measure wrist temperature and forehead temperature. However, these devices have unproven reliability [15].

Many studies compare temperature measurements for different parts of the human body using different thermometers. Most of the forehead thermometers are contact-type. The measurements of body temperature depend on the type of thermometer, the sensing elements and the manufacturers’ claimed performance for each thermometer.

This study uses two commercial tympanic IRTs and an industrial IRT to measure the tympanic, forehead and wrist temperatures, and the measurement data is analyzed using statistical techniques. The results for the measurement of body temperature are used to define the threshold for fever screening using a forehead IRT.

## 2. Equipment and Methods

### 2.1. Infrared Thermometer

The received energy from the measuring target is as follows:

(1) $$E=\varepsilon \sigma \;T_{k}^{4}$$

σε is the Stefan–Boltzmann constant, ε is the emissivity of this target and *T_k_* is the absolute temperature of this target in *K*.

If the reflection of the ambient radiation and the self-radiation of the infrared thermometer are considered, Equation (1) is revised as follows:

(2) $$E_{2}=C_{1}\left[\varepsilon T_{k}^{4}+\left(1-\varepsilon \right){T_{amb}}^{4}-{T_{dev}}^{4}\right]$$

where *C*_1_ is the constant of infrared device, *T_amb_* is the ambient temperature in *K* and *T_dev_* is the device temperature in *K*.

If the infrared thermometers do not cover all ware length range, an exponent constant, *n* is used to replace the power constant of 4.

(3) $$E_{3}=C_{1}\left[\varepsilon T_{k}^{n}+\left(1-\varepsilon \right){T_{amb}}^{n}-{T_{dev}}^{n}\right]$$

The target temperature is then determined as follows:

(4) Tk={[E3−C1(1−ε)Tambn−C Tdevn]C1·ε}1n

To ensure the accuracy of the $T_{k}$ measurement, the $T_{dev}$ value must be detected with a thermometer of highly accuracy and the *T_amb_* is kept stable.

Two types of tympanic IRTs and one industrial IRT were used for this study: a BRAUN IRT-3020 Thermoscm model (Braun Co., Melsungen, Germany), an OMRON MC-510 Gentle Temp model (OMRON Co., Kyoto, Japan) and a THI 301 IRT (Tasco Co., OSAKA, Japan). The specifications of these IRTs are listed in Table 1.

### 2.2. Standard Temperature for Calibration

The temperature calibrator that is used for this study is a TC-2000 Scan Sense (Instrutek Co., Larvik, Norway). The temperature of an oil bath was detected using a PT-100 thermometer and the manufacturer’s specification signifies an uncertainty of 0.03 °C.

The IRTs were calibrated using theTC-2000 temperature calibrator at temperatures of 34 °C, 36 °C and 38 °C. The calibration methods are described in a previous study [49,50].

### 2.3. Experimental Design

Two experiments were performed for this study.

#### 2.3.1. The First Experiment

The BRAUN IRT-3020 thermometer was used to measure the tympanic temperature in the right and left ears, the forehead temperature and the wrist temperature of the same subject.

The test used 614 males and females, aged from 16 to 60 years.

The ambient temperature was measured with a PT-100 hand-held thermometer (Electronic Temperature Instrument Ltd., West Sussex, UK). The accuracy of this thermometer is ±0.1 °C.

The temperature of the measurement room was different in relation to its air-conditioning devices and the testing time of day. All subjects waited in the room for five minutes after entrance and before measurement.

#### 2.3.2. The Second Experiment

For the same subject, The BRAUN IRT-3020 thermometer and the OMERN MC-510 thermometer were used to measure the tympanic temperature in both ears. The THI-301 thermometer was used to measure the temperature at the left and right wrists and the forehead temperature.

Due to the different periods of experiments, the subjects of the first and second experiments were different. 168 individuals aged 18–42 years were used for this test.

### 2.4. Statistical Analysis

All temperature readings were analyzed using Excel software. Data are expressed as a mean and a standard deviation. The coefficient of variance (CV) value is calculated to determine the accuracy of measurement.

CV = (standard deviation)/mean(5)

Significant differences between the two sets of data were tested using a paired t-test. A *p* value equal to or less than 0.05 denotes statistical significance. The relationship between measurement values is determined using a correlation analysis and a regression analysis.

## 3. Results

### 3.1. The First Experiment

Six hundred fifty-nine randomly selected citizens were recruited for this study. The results of the tests are listed in Table 2. The CV value is less than 1.0% for tympanic temperature. The CV value for forehead is 1.129%, and for wrist temperature is 1.332%. A CV of 5% indicates good precision [51]. The distribution for forehead and wrist temperature is higher than for tympanic temperature. The correlation between these measurement values is shown in Table 3. The correlation coefficients are small, except for the measurement values for two ears.

A paired t-test for the tympanic temperature in both ears showed that there is no significant difference (t = 0.474, *p* < 0.05) in the readings for tympanic temperature for left and right ears.

**Figure 1:**
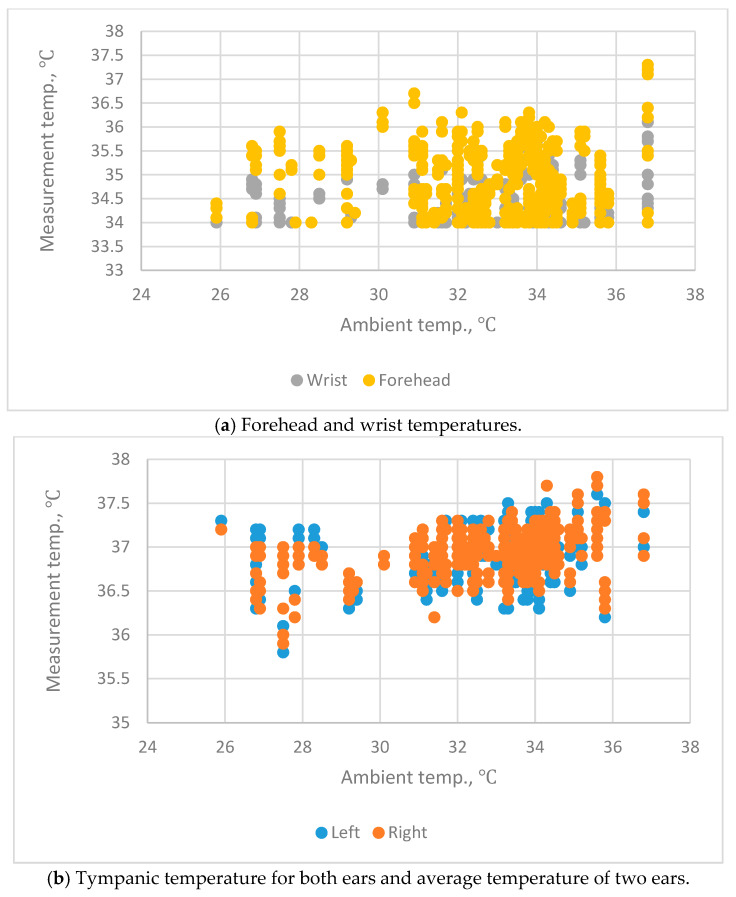
The relationship between the air temperatures and body temperatures measured at the ear, forehead and wrist.

The relationship between the air temperature and the body temperature as measured at the ear, the forehead and the wrist are shown in Figure 1. There is no significant correlation between air temperature and body temperature. That is, the environmental temperature does not have a significant effect on the body temperature measurement.

The sensing element of BRAUN IRTs is pyroelectrical detectors, and the uncertainty of this device is low [49], so the average of the left and right tympanic temperature (Ear_ave_) is denoted as the standard body temperature.

**Figure 2:**
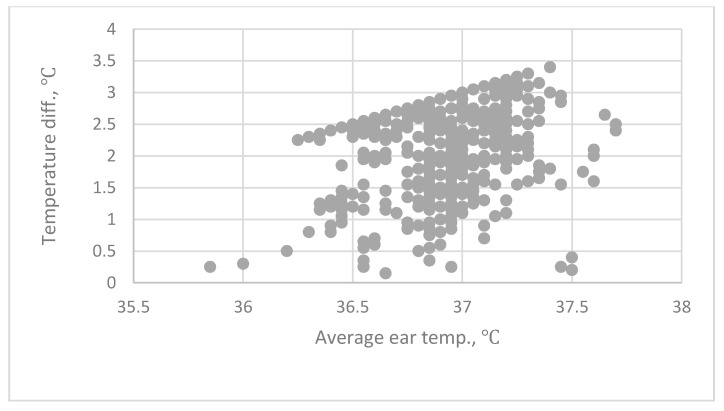
The relationship between Ear_ave_–forehead and Ear_ave_ (the average temperature of both ears). Ear_ave_–forehead is the difference between average ear temperature (Ear_ave_) and forehead temperature.

**Figure 3:**
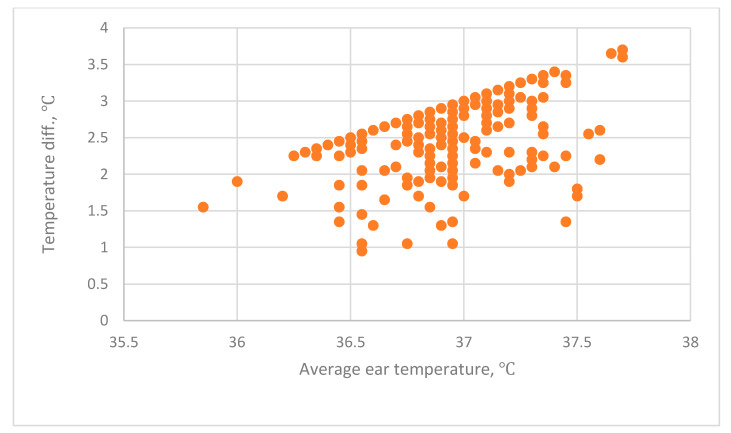
The relationship between Ear_ave_–wrist and Ear_ave_ (the average temperature of both ears). Ear_ave_–wrist is the difference between the average ear temperature (Ear_ave_) and wrist temperature.

This experiment used a tympanic IRT to directly measure the forehead and wrist temperatures. The different between Ear_ave_ and the forehead is termed Ear_ave_–forehead and that between Ear_ave_ and the wrist temperature is termed Ear_ave_–wrist. The relationship between Ear_ave_–forehead and Ear_ave_ is shown in Figure 2. The relationship between Ear_ave_–wrist and Ear_ave_ is shown in Figure 3. The lowest possible reading for the BRAUN thermometer is 34 °C, so some of the data in the two figures show a fixed pattern.

This device cannot measure a temperature of less than 34 °C. A statistical correlation analysis and regression analysis shows that there is no significant correlation between tympanic temperature and forehead and wrist temperature. The mean forehead temperature is 34.71 °C ± 0.392 °C and the mean wrist temperature is 34.16 °C ± 0.355 °C. The mean difference between the average tympanic temperature and the forehead temperature is 2.2 °C ± 0.411 °C. These results are similar to those of a previous study [18,52,53].

### 3.2. The Second Experiment

The second experiment measured the tympanic temperature in the left and right ears using a BRAUN and an OMRON IRT, and the temperature of the forehead and two wrists was measured using an industrial THI-301 IRT for the same subject at the same time.

The results of the measurements are listed in Table 4. A paired t-test for the temperature in the left and right ears gives a value of t = 2.38 (*p* < 0.02) for the BRAUN IRT and of t = 2.48 (*p* < 0.02) for the OMRON IRT. The paired t-test shows that there is no significant difference at the level of *p* < 0.02. The difference in the readings of the two IRTs is 0.22 °C ± 0.201 °C, which is acceptable for practical applications.

The measurement results for the THI-301 IRT are listed in Table 5. The CV values for the three measurements are lower than 2%. The criterion for dispersion is 5% [51]. These CV values demonstrate that the device has good precision. The minimum respective values for the forehead, left wrist and right wrist temperature are 33.8 °C, 32.4 °C and 32.2 °C. The tympanic IRT cannot read a temperature of less than 34 °C so the tympanic IRT is not sufficiently accurate at this temperature to measure the wrist temperature.

The BRAUN IRT has the lowest values of combined uncertainty [52], so the average value for the right and left tympanic temperatures as measured using the BRAUN IRT (noted as BRAUN_ave_) are served as the standard values for this study. The results are listed in Table 6.

The difference between the average temperature that is measured by the BRAUN and the temperature at the forehead is denoted as BRAUN_ave_–Forehead and is 2.107 °C ± 0.301 °C. The difference between the average temperature that is measured by the BRAUN and the temperature at the right wrist is denoted as BRAUN_ave_–Wrist_right_ and is 3.2781 °C ± 0.527 °C. The difference between the average temperature that is measured by the BRAUN and the temperature at the left wrist, which is denoted as BRAUN_ave_–Wrist_left_, is 3.308 °C ± 0.534 °C. The mean forehead temperature is 2.017 °C lower than the mean tympanic temperature, and the mean wrist temperature is 3.3 °C lower than the mean tympanic temperature. The paired t-test for the wrist temperatures of the left and right hands, measured using a THI-301 industrial IRT, is insignificant (t = 0.71, at *p* < 0.05 criterion). A study by Werner et al. [53] showed that the difference between the core temperature and the forehead temperature is 2.1 °C–2.3 °C and the difference between the core temperature and the wrist temperature is 3.0 °C–3.3 °C. The result of this study is similar to that of the previous study [53]. The correlation analysis for the measurement results for the second experiment is shown in Table 7. All correlation coefficients for the observed values are low, except for the temperatures in left and right ears which were detected using two tympanic IRTs.

**Figure 4:**
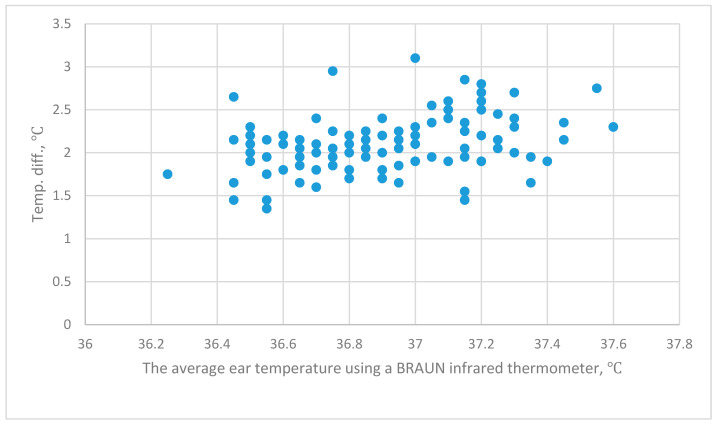
The relationship between B_ave_–Forehead and B_ave_: B_ave_–Forehead is the difference between the average era temperature (Ear_ave_) using a BRAUN infrared thermometer and the forehead temperature using a THI-301 infrared thermometer.

**Figure 5:**
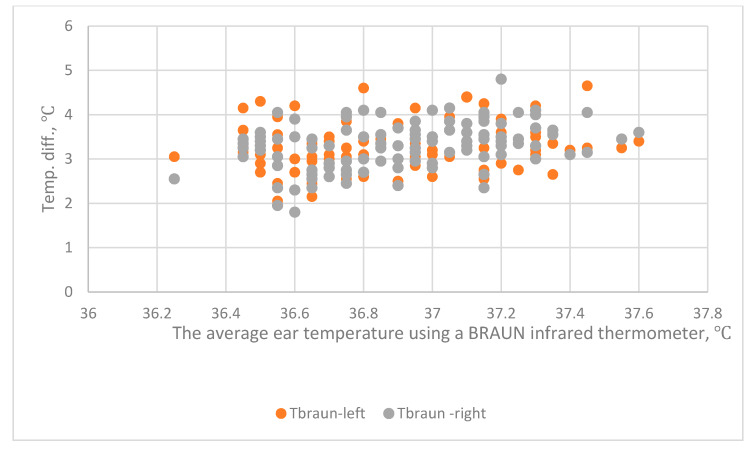
The relationship between the B_ave_–Wrist_right_, the B_ave_–Wrist_left_ and the B_ave_. B_ave_–Wrist_right_ is the difference between the average ear temperature (Ear_ave_) using a BRAUN infrared thermometer and the right wrist temperature using a THI-301.

The relationship between BRAUN_ave_–Forehead and BRAUN_ave_ is shown in Figure 4. The relationships between BRAUN_ave_–Wrist_right_, BRAUN_ave_–Wrist_left_ and BRAUN_ave_ are shown in Figure 5. Correlation analysis and regression analysis show that there is no significant correlation.

## 4. Discussion

These results show the diversity of the body temperature. There is a high correlation between the tympanic temperature in the left and right ears. However, a paired t-test shows different results for the two experiments. In the first experiment, there is no significant difference between the temperature in the right and left ears, as measured using a BRAUN IRT (*p* < 0.05). In the second experiment, there is a significant difference in the temperature in the right and left ears for the two types of IRT (*p* < 0.002). The subjects for the two experiments were different. The first experiment involved 659 subjects and the second experiment involved 114. The size of the sample and a different age distribution affects the standard deviation, which affects the calculation of t values.

In the first experiment, the difference between the mean tympanic temperature and the forehead temperature is 2.20 °C ± 0.711 °C. This experiment used a tympanic IRT to detect the forehead temperature. The emissivity of this device cannot be adjusted. In the second experiment, the difference between the mean tympanic temperature and the forehead temperature is 2.1 °C ± 0.301 °C. This experiment used an industrial IRT to measure the forehead temperature. The emissivity of this industrial device was adjusted to 0.98. Both experiments produce similar results. The forehead temperature is 2.1 °C or 2.2 °C lower than the tympanic temperature. In the second experiment, the wrist temperature that is measured using a THI-301 IRT is 3.3 °C lower than the tympanic temperature. These measurements for wrist temperature do not represent the actual body temperature, so measurements of the wrist temperature cannot be used to establish a threshold for screening for fever.

Measurements of forehead temperature are convenient, fast and involve a low risk of infection, so they are used to screen for fever to identify possible victims of the novel coronavirus (COVID-19). However, there is no international standard for the threshold of forehead temperature that indicates fever. The Department of Health of Hong Kong [54] recommended a threshold level for fever of >38 °C for ear temperature and >36 °C for forehead temperature. The difference between the two critical temperatures is 2 °C. The results of this study are close to these figures.

Liu et al. [12] determined the accuracy of alternative IR techniques for fever screening during the 2003 SARS epidemic. Two types of IRTs were used to measure tympanic and forehead temperature. The results for 276 subjects showed a tympanic temperature of 36.44 °C ± 0.37 °C and a forehead temperature of 35.63 °C ± 0.36 °C. Ng et al. [13] measured forehead temperature for 1000 healthy subjects using three handheld IRTs. The temperature was measured as 33.3 °C ± 1.18 °C using IR thermometers, but the results of the three IR thermometers were inconsistent. Their study noted that a forehead temperature of more than 35.5 °C suggests fever. Ng et al. [14] determined that the forehead and tympanic temperature for 567 patients, measured using an IRT, differs by 2.34 °C. However, the study does not report the specifications of the sensors or the calibration technique. Williams et al. [55] measured tympanic and forehead temperature using different IRTs. The tympanic was measured as 36.7 °C ± 0.5 °C and the forehead temperature as 34.0 °C ± 0.7 °C, and they concluded that measurements of forehead temperature are not as representative of the actual body temperature as tympanic temperature. In our study, the tympanic and forehead temperatures measured by BRAUN IRT were 36.9 °C ± 0.286 °C and 34.714 °C ± 0.392 °C, respectively. The results of our study were similar to those of Ng et al. [14].

There is inconsistency in the results of studies. Ganio et al. [56] and Edling et al. [57] reported that a forehead IRT has good repeatability but cannot be applied as a clinical device because there is a fixed offset. Langham et al. [39] reported a 2 °C difference in forehead temperature, as measured using an IRT, and the reference value was the bladder temperature. Chiappini et al. [58] and Teran et al. [59] determined that the use of an IRT to measure forehead temperature gives accurate results, so these are suited to clinical use. However, the fixed offset could be explained as the physiological phenomenon explained by the different temperature distribution between the inner and surface forehead skin. The precision of forehead IRT could be evaluated with the CV values in our study.

The mean difference between a tympanic IRT and a contact-type deep skin forehead thermometer was less than 1 °C [27,28,34]. If an IRT is used to measure the forehead temperature, the surface temperature of the skin is measured, not the muscle temperature near the superficial temporal artery [47]. Core body temperature is the standard temperature of the body. It is defined as the temperature of the blood perfusing the thermoregulatory receptor in the hypothalamus [4]. The tympanic membrane has the same vascular supply that perfuse the hypothalamus, so it is the best site to measure the core body temperature [60].

The superficial temporal is a side drench of the carotid artery and feeds beneath the vascular bed of the head’s skin [47]. If a contact type thermometer is used to measure the forehead temperature, the sensing element, which is a thermistor, must touch the skin of the forehead. The sensors must be inserted into the muscle. The effect of the environment, such as wind speed, is neglected, so the measurement of forehead temperature using a contact-type thermometer is close to the tympanic temperature [33,34]. However, the fixed difference between the measurement values of tympanic and forehead IRTs needs to be considered.

A tympanic IRT is painless in use, does not involve contact with the membrane and gives a rapid result. However, the cover must be frequently replaced and the probe position must be adjusted to reduce distress to subjects. The measurement of forehead temperature using an IRT is more convenient than that of tympanic IRT.

The performance of an IRT is affected by the calibration method and environmental factors, such as solar radiation and wind speed over the skin [61,62]. Standard calibration practices [6,7,62,63] and proper operational procedures ensure the accuracy of the measurement of the forehead temperature.

The critical tympanic temperature that is used to screen for fever is 38 °C [1,54]. The Hong Kong government’s threshold level for fever is 38 °C for tympanic temperature and 36 °C for forehead temperature. Ng et al. [13,14] recommended a temperature of 35.5 °C to screen for fever. Previous studies show that the mean difference between forehead and tympanic temperature is 1.9 °C [64]; 2.7 °C [55]; and 2.0 °C [39]. This study determines that the mean difference between tympanic temperature and forehead temperature is 2.2 °C.

The uncertainty of three IRTs was calculated by Chen et al. [55]. At 36 °C, the combined uncertainty for the BRAUN is 0.15 °C, for the OMERO is 0.23 °C and for the THI-301 is 0.29 °C. This study determines that the tympanic temperature that is measured by the BRAUN IRT for the two experiments is 36.9 °C ± 0.286 °C and 36.937 °C ± 0.301 °C. The forehead temperature that is measured by the BRAUN is 34.714 °C ± 0.392 °C and by the THI-301 IRT is 34.80 °C ± 0.343 °C. The difference temperature between tympanic and forehead temperature for the two experiments is 2.20 °C ± 0.411 °C and 2.107 °C ± 0.311 °C. The difference between tympanic temperature and forehead temperature ranges from 2.1 °C to 2.2 °C for the two experiments.

The threshold for fever level for tympanic temperature is 38 °C. The measurement uncertainty for an IRT means that the threshold for fever for forehead temperature that is measured using an IRT could be 36 °C. Therefore, forehead temperature does not give as accurate a representation of actual body temperature as the tympanic temperature, and as such, forehead IRTs are suitable for quick screening but are not as accurate as a traditional body temperature thermometer.

The combined uncertainty of two IRTs at 36 °C for the BRAUN and the THI-301 was 0.42 °C and 0.45 °C, respectively. For the BRAUN IRT, the mean forehead temperature was 34.714 °C. The 95th and 99th percentiles were 35.40 °C and 35.69 °C, respectively. For the THI-301 IRT, the mean forehead temperature was 34.802 °C. The 95th and 99th percentiles were 35.54 °C and 35.85 °C, respectively.

If the 35.5 °C is selected as the threshold value for fever screening, the percentiles are 96.94% and 93.96% for BRAUN IRT and THI IRT, respectively. That is, there could be a false-positive region of 3.06% or 6.04%, respectively, and this will cause trouble in the fever screening of the masses. If the 36.0 °C is selected as the threshold value for fever screening, the percentiles are 96.89% and 99.61% for BRAUN IRT and THI IRT, respectively and the region of false are small.

Considering the measurement uncertainty of IRTs and the requirement of practical operation (ease of use, speed and convenience), the standard operating procedure (SOP) is proposed to screen for fever using measurements of forehead temperature.

- Tympanic and forehead IRTs should be used.
- Both IRTs must be calibrated using a black box method to ensure accuracy [6,7,47,63].
- The threshold level for fever is 36.0 °C for a forehead temperature measurement using an IRT.
- If the forehead temperature of a subject is measured to be higher than 36.0 °C, the tympanic temperature must be measured.
- If the tympanic temperature is >38 °C. The subject is considered to have a fever condition and clinical treatment is advised.

## 5. Conclusions

Forehead temperature measurement using an infrared thermometer is used to rapidly screen for fever to identify victims of the coronavirus (COVID-19). The performance of this type of thermometer and the threshold temperature for screening for fever is studied. This study uses two medical tympanic IRTs and an industrial thermometer IRT to measure the tympanic temperature in both ears and the temperature at the wrist and forehead.

The results show that the tympanic temperature that is measured using the BRAUN IRT is 36.9 °C ± 0.286 °C and 36.937 °C ± 0.301 °C for the two experiments. The forehead temperature that is measured by the BRAUN is 34.714 °C ± 0.392 °C and by the THI-301 IRT is 34.80 °C ± 0.343 °C. The temperature differences between the tympanic temperature and the forehead temperature for the two experiments are 2.20 °C ± 0.411 °C and 2.107 °C ± 0.311 °C, respectively. The difference between the tympanic temperature and the forehead temperature ranges from 2.1 °C to 2.2 °C for the two experiments.

The measurement uncertainty for an IRT means that the threshold for fever when measuring forehead temperature is 36 °C. This study proposes a standard operating procedure (SOP) to screen for fever by measuring forehead temperature using an IRT. Forehead IRTs are suited to quick screening but are not be used to represent the actual body temperature as tympanic temperature measurements.

## Figures and Tables

**Table 1:** Specifications of the infrared thermometers.

	BRAUN IRT-3020	OMEON MC-510	Tasco THI-301
Sensing elements	Pyroelectrical detector	Thermopile	Thermopile
Measuring range	34 °C–42.2 °C	34 °C–42.2 °C	0 °C–50 °C
Resolution	0.1 °C	0.1 °C	0.1 °C
Nonlinearity and repeatability	37.0 °C–39.0 °C, ±0.1 °C	36.0 °C–39.0 °C, ±0.2 °C	±0.5 °C
	≤37.0 °C or ≥39 °C, ±0.2 °C	≤37.0 °C or ≥39 °C, ±0.3 °C	After calibration

**Table 2:** Measurements of the tympanic temperature, forehead temperature and wrist temperature using a BRAUN infrared thermometer.

	Ear_left_	Ear_right_	Forehead	Wrist	Ear_ave_	Ear_diff_	Ear_ave_–Forehead	Ear_ave_–Wrist	Forehead–Wrist
Mean	36.904	36.912	34.714	34.1644	36.911	−0.258	2.1966	2.7467	0.5501
Standard deviation	0.286	0.259	0.392	0.455	0.264	0.139	0.411	0.415	0.532
CV (%)	0.775	0.712	1.129	1.332					
Min	35.8	35.9	34.0	34.0	35.85	−0.3	0.15	0.95	0
Max	37.5	37.8	37.3	36.1	37.7	0.4	3.4	3.7	2.1

Note: 1. Ear_left_: the reading values for the left ear. 2. Ear_right_: the reading values for the right ear. 3. Ear_ave_: the average of reading values for both ears. 4. Ear_diff_: the difference between the measurements for two ears. 5. Ear_ave_–Forehead: the difference between the average tympanic temperature and forehead temperature. 6. Ear_ave_–Wrist: the difference between the average tympanic temperature and wrist temperature. 7. Forehead–Wrist: the difference between the forehead and wrist temperature. 8. CV: coefficient of variance, CV = (standard deviation)/mean.

**Table 3:** The correlation between measurement results for the first experiment.

	Air Temp	Ear_left_	Ear_right_	Forehead	Wrist
Air temp	1				
Ear_left_	0.2627	1			
Ear_right_	0.3958	0.8602 *	1		
Forehead	−0.018	0.1004	0.1488	1	
Wrist	0.0982	0.0656	0.1313	0.1005	1

Note: * represented the relationship is significant at *p* < 0.05 according to Duncan’s multiple range test.

**Table 4:** Statistics of the measure results for tympanic temperatures for the second experiment.

	B_right_	B_left_	B_ave_	B_diff_	O_right_	O_left_	O_ave_	O_diff_
Mean	36.937	36.881	36.909	0.0461	36.740	36.679	36.719	0.0403
Standard deviation	0.301	0.293	0.286	0.155	0.264	0.266	0.250	0.174
CV (%)	0.815	0.794			0.719	0.725		
Min	36.3	36.2	36.25	0.3	36.2	36.0	36.1	−0.5
Max	37.7	37.5	37.6	0.4	37.2	37.4	37.25	0.4

Note: 1. B_right_: the reading values for the right ear using a BRAUN tympanic infrared thermometer. 2. B_left_: the reading values for the left ear using a BRAUN tympanic infrared thermometer. 3. B_ave_: the average of the right and left temperatures using a BRAUN tympanic infrared thermometer. 4. B_diff_: the difference between the right and left temperature using a BRAUN tympanic infrared thermometer. 5. O_right_: the reading values for the right ear using an OMRON tympanic infrared thermometer. 6. O_left_: the reading values for the left ear using an OMRON tympanic infrared thermometer. 7. O_ave_: the average of the right and left temperatures by OMRON tympanic infrared thermometer. 8. O_diff_: the difference between the right and left temperatures using an OMRON tympanic infrared thermometer.

**Table 5:** The statistics of the measurement results for a THI-301 thermometer.

	Forehead	Wrist_right_	Wrist_left_
Mean	34.802	33.601	33.605
Standard deviation	0.343	0.602	0.554
CV (%)	0.986	1.791	1.640
Min	33.8	32.4	32.2
Max	35.7	34.8	34.7

1. Wrist_right_: the right wrist temperature; 2. Wrist_left_: the left wrist temperature; 3. CV: Coefficient of variance, CV = (standard deviation)/mean.

**Table 6:** The statistics of the comparison of measurement.

	B_ave_–Forehead	B_ave_–Wrist_right_	B_ave_–Wrist_left_	Wrist_right_–Wrist_left_
Mean	2.1070	3.2781	3.3035	0.02544
Standard deviation	0.311	0.527	0.534	0.385
Min	1.32	1.8	2.05	−0.9
Max	3.10	4.8	4.65	0.9

B_ave_–Forehead: the difference between the average tympanic temperatures using a BRAUN tympanic infrared thermometer and the forehead temperatures using a THI-301 infrared thermometer. B_ave_–Wrist_right_: the difference between the average tympanic temperatures using a BRAUN tympanic infrared thermometer and the right wrist temperatures using a THI-301 infrared thermometer. B_ave_–Wrist_left_: the difference between the average tympanic temperatures using a BRAUN tympanic infrared thermometer and left wrist temperatures using a THI-301 infrared thermometer. Wrist_right_–Wrist_left_: the difference between the wrist temperatures of two hands using a THI-301 infrared thermometer.

**Table 7:** The correlation for the measurements for the second experiment.

	B_right_	B_left_	O_right_	O_left_	THI_forehead_	THI_right wrist_	THI_left wrist_
B_right_	1						
B_left_	0.864	1					
O_right_	0.670	0.617	1				
O_left_	0.693	0.668	0.784	1			
THI_forehead_	0.424	0.472	0.191	0.246	1		
THI_right wrist_	0.206	0.167	0.075	0.041	0.448	1	
THI_left wrist_	0.3526	0.279	0.0518	0.0930	0.4758	0.738	1

B_right_: the reading values for the right ear using a BRAUN tympanic infrared thermometer. B_left_: the reading values for the left ear using a BRAUN tympanic infrared thermometer. O_right_: the reading values for the right ear using an OMRON tympanic infrared thermometer. O_left_: the reading values for the left ear using an OMRON tympanic infrared thermometer. THI_forehead_: the reading values for the forehead using a THI-301 infrared thermometer. THI_right wrist_: the reading values for the right hand using a THI-301 infrared thermometer. THI_left wrist_: the reading values for the left hand using a THI-301 infrared thermometer.
